# Dialysis Efficiency of AN69, a Semisynthetic Membrane Not Well Suited for Diffusion

**DOI:** 10.5402/2013/185989

**Published:** 2012-12-20

**Authors:** M. E. Herrera-Gutiérrez, G. Seller-Pérez, D. Arias Verdu, C. Jironda-Gallegos, M. Martín-Velázquez, G. Quesada-García

**Affiliations:** ^1^ICU, Carlos Haya Hospital, Carlos Haya Avenue s/n, 29010 Málaga, Spain; ^2^Nephrology, Carlos Haya Hospital, 29010 Málaga, Spain

## Abstract

AN69 membrane is not suited for diffusion, with an suggested limit at 25 mL/min dialysate flow rate. When prescribing continuous hemodialysis this threshold must be surpassed to achieve. We designed a study aimed to check if a higher dose of dialysis could be delivered efficiently with this membrane. Ten ICU patients under continuous hemodiafiltration with 1.4 m^2^ AN69 membrane were included and once a day we set the monitor to exclusively 50 mL/min dialysate flow rate and 250 mL/min blood flow rate and after 15 minutes measured dialysate saturation for urea, creatinine, and *β*_2_-microglobulin. We detected that urea saturation of dialysate was nearly complete (1.1 ± 0.09) for at least 40 hours, while creatinine saturation showed a large dispersion (0.86 ± 0.22) and did not detect any relation for these variables with time, blood flow, or anticoagulation regime. Saturation of *β*_2_-microglobulin was low (0.34 ± 0.1) and decreased discretely with time (*r* ^2^ = 0.15, *P* < 0.05) and significantly with TMP increases (*r* ^2^ = 0.31, *P* < 0.01). In our experience AN69 membrane shows a better diffusive capability than previously acknowledged, covering efficiently the range of standard dosage for continuous therapies. Creatinine is not a good marker of the membrane diffusive capability.

## 1. Introduction

Continuous renal replacement therapies (CRRT) have changed substantially the last two decades. Developed as a practical method to treated acute kidney failure in unstable patients and based in the use of convection, in the first stages a low solute clearance capability was characteristic and subsequent changes (as an early shift from an arterial-vena to a vena-vena approach) [1] were aimed to improve performance. To raise the clearance of uremic toxins, CRRT procedures evolved to slightly different methods like continuous hemodiafiltration (CHDF) [2] or continuous hemodialysis (CHD) [3] because a supplementary diffusive transport can improve the clearance of low molecular weight toxins, such as urea [4]. 

Dosage delivered as convective treatment can theoretically be raised without limits but in the real practice we have a limiting factor, that is, blood flow. When this limit has been reached to augment the dialysate flow seems an attractive alternative but some early reports demonstrated that when we set the dialysate over 25 mL/min, efficiency of the treatment is seriously compromised and this effect is related to the membrane involved [5].

In our unit, the weaning from CRRT is usually performed with slow intermittent dialysis delivered with the same CRRT monitor, in sessions lasting 10–12 hours. During this weaning phase some patients require a dose of dialysis theoretically surpassing the capability of the AN609 membrane. We designed this study to detect whether high dose dialysis performed with a membrane of low diffusive capability (AN69) was able to fulfill efficiently the requirements for our patients.

Our objective was to determine if a higher dose of dialysis could be delivered efficiently with this membrane.

## 2. Methods

We conducted a prospective observational study, collecting data from 10 ICU patients with acute kidney injury managed with CHDF.

### 2.1. CRRT Protocol

CHDF is conducted in our unit with a Prismaflex monitor (Hospal) and a 1.4 m^2^ AN69 membrane filter (HF-1400, Hospal). Bicarbonate buffered fluid is infused in postdilution mode and the anticoagulant regime is adjusted to the clinical condition of the patient [6], using three alternatives: nonfractionated heparin at 5 U/Kg/h, epoprostenol at 5 ngr/Kg/min, or no anticoagulation. Initial prescribed dose is 35 mL/Kg/h, with the convective component as high as possible (according to catheter performance and keeping filtration fraction below 20%); when the complete dose cannot be achieved the rest is delivered as dialysis. When the patient is hemodynamic stable the dose is lowered, aiming for internal milieu normality, and usually convective and diffusive components are equaled. Finally, the weaning process is performed with slow intermittent dialysis delivered with the Prismaflex monitor in sessions lasting 10–12 hours every day. During the weaning phase of the treatments some patients require a dose of dialysis theoretically surpassing the capability of the AN609 membrane. So we designed this protocol to assure that an efficient treatment was delivered.

### 2.2. Measurements

Every day and during the morning shift, treatment prescription was changed temporarily to a blood flow (*Q*_*b*_) of 250 mL/min, a dialysate flow rate (*Q*_*d*_) of 50 mL/min (3 L/h), and zero ultrafiltration/zero negative balance; anticoagulation was maintained without changes. The monitor was kept running for 15 minutes before samples were taken and then was immediately reverted to the original prescription for each patient. Transmembrane pressure (TMP) and anticoagulation regime at this time were registered for each measure.

Blood samples were taken pre- and postfilter using the ports in the circuit and a sample of the effluent was extracted from the port in the effluent line. Samples were transferred immediately to the laboratory for determination of urea, creatinine, and *β*_2_-microglobulin.

Saturation of the dialysate was calculated using the equation:

(1) $$\begin{array}{c} \text{Saturation}=\;\left(\frac{\left[E_{f}\right]}{\left(\left[I_{n}\right]+\left[O_{u}\right]\right)/2}\right), \end{array}$$

where [*E*_*f*_] is the concentration of a solute in the effluent, [*I*_*n*_] is the concentration in the before filter blood sample, and [*O*_*u*_] is the concentration in the after filter sample.

### 2.3. Statistical Analysis

 Data are shown as mean (standard deviation) or *n* (percentage). To test hypothesis we used *U* Mann-Whitney or Kruskal-Wallis, and for the study of the relationship between continuous variables a linear regression analysis was performed. A 0.05-signification level was used for all tests. 

## 3. Results

We performed 44 measurements in 10 patients, with a median of 3 measures/patient (in 3 patients 1 measure and in one 16 measures).

All patients were treated with bicarbonate buffered hemodiafiltration with a mean *Q*_*b*_ of 241 ± 37.1 mL/min and a mean dose (*Q*_*d*_) of 2506  ±  69 mL/h. Mean TMP at the moment of the measurement was 81 ± 60 mmHg and the anticoagulant regime employed at this point was heparin in 18 (40.9%), epoprostenol in 9 (20.5%), and none in 17 (38.6%) measures. 

**Figure 1:**
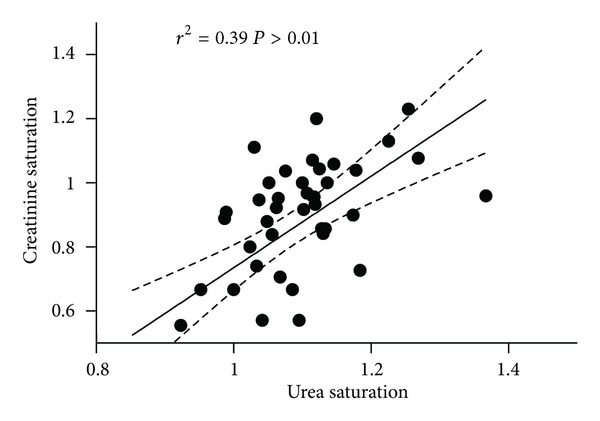
Relationship between urea and creatinine saturation of dialyzer.

Mean saturation of dialysate was 1.08 ± 0.09 for urea and 0.86 ± 0.22 for creatinine (*P* < 0.001), and the relationship between both measures showed a wide distribution of data (Figure 1). Mean saturation of *β*_2_-microglobulin was 0.34 ± 0.12.

**Figure 2:**
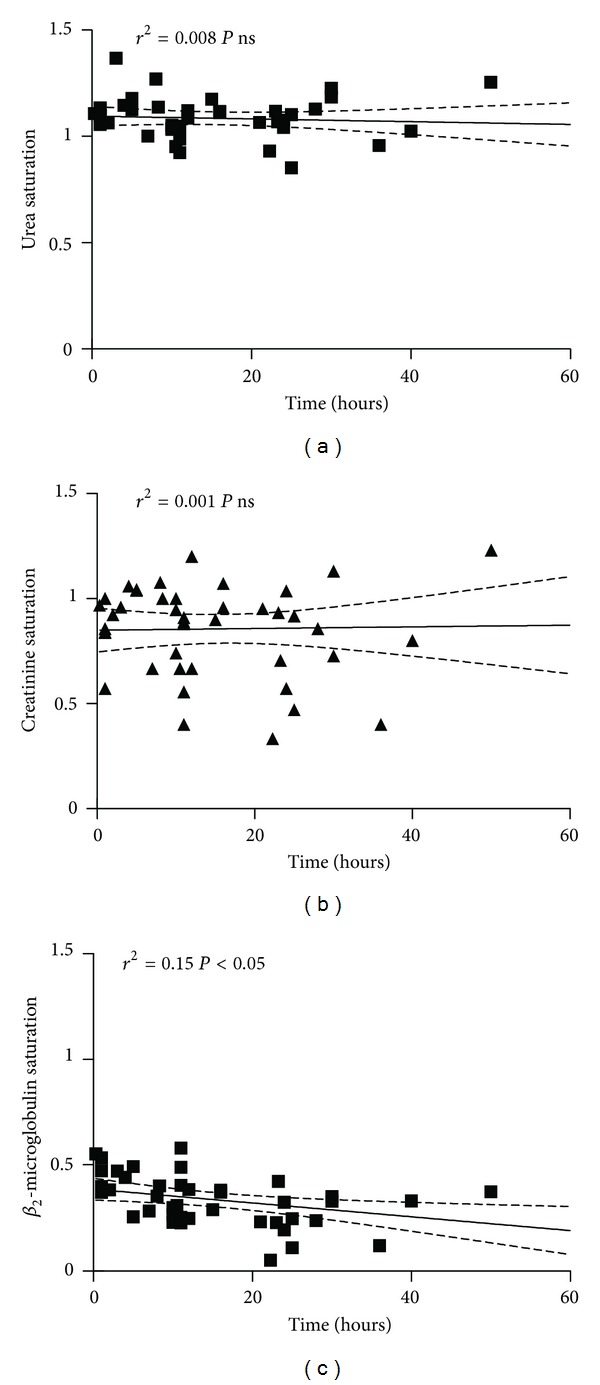
Relation between saturation of dialyzer and filter running time for urea, creatinine, and Beta_2_-microglobulin.

**Figure 3:**
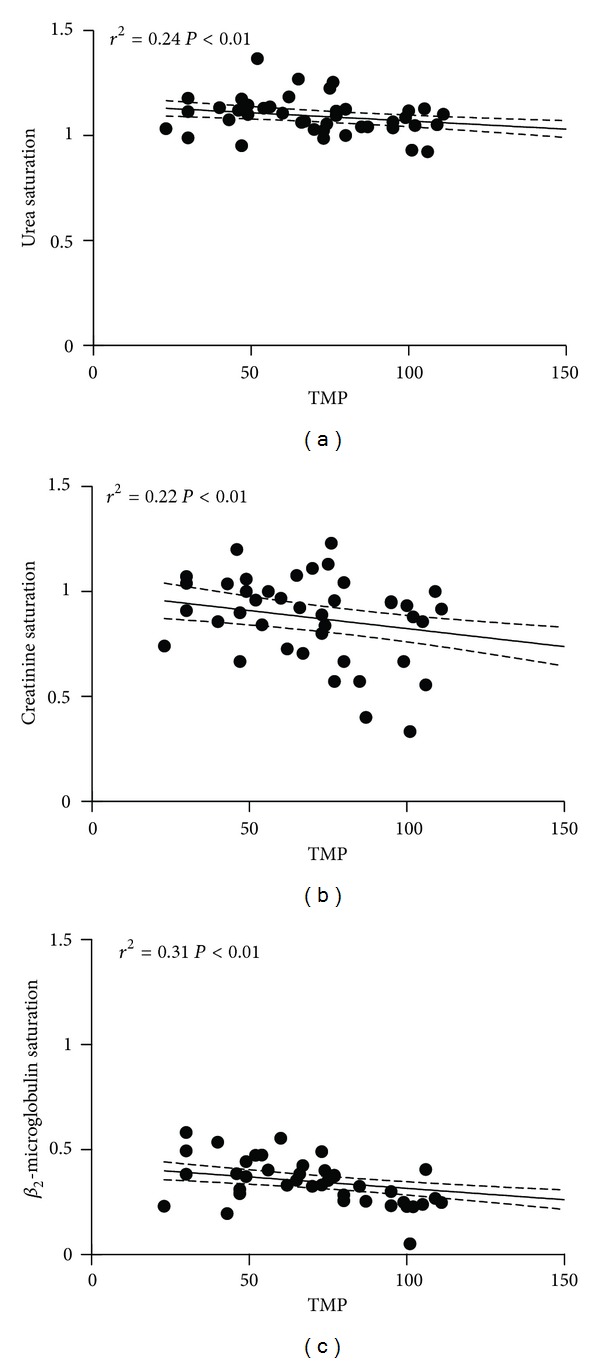
Relation between saturation of dialyzer and transmembrane pressure (in mmHg) for urea, creatinine, and Beta_2_- microglobulin.

We detected a decrement of *β*_2_-microglobulin in relation to filters running time (*r* ^2^ = 0.15, *P* < 0.05) and not for urea or creatinine but, while urea saturation was close to 1 without evident changes in time, creatinine saturation showed an erratic behavior with a wide dispersion of data (Figure 2). A fall in dialysate saturation following an increment of TMP was observed for the three molecules, more marked for *β*_2_-microglobulin than urea or creatinine and once more with a wide dispersion for the last one (Figure 3). The anticoagulant regime employed did not interfer with the results (Table 1).

## 4. Discussion

Dose prescription in CRRT has risen steadily since the appearance of these therapies. The best dose is an open debate [7–10] but in practice is markedly higher than two decades ago. Other aspect under debate is whether this dose must be delivered as convection, diffusion, or both but the most widely employed modality is mixed CHDF [11–13]. When prescribing CRRT, synthetic membranes with low diffusive performance are employed and a raise in the dialysate flow rate will not be always followed by a proportional increment in dose. As a general rule, keeping dialysate flow rate below 25–30 mL/min has been considered adequate [14, 15] but our results demonstrate that with a 1.4 m^2^ AN69 membrane this threshold can be raised up to 50 mL/min (3 L/h) while keeping dialysate saturation in the optimal range.

When prescribing CRRT, high permeability synthetic membranes are used and some of these membranes show a low performance in diffusion [16]. The classical membrane requirement to be met with respect to diffusive clearance has been in the past the complete saturation of the dialysate at flow rates up to 30 mL/min [5] because beyond this boundary the ability to completely saturate the dialysate begins to fall [16]. This effect is potentially more significant when using polyamide compounds, which show low diffusive transport properties [17]. Now we can confirm that a complete saturation of the dialysate should be expected with flows up to 50 mL/min (3 L/h) (nearing the dose achieved with other diffusive treatments like SLEDD) [18–20]. 

Interrelation between convection and diffusion in the ultrafiltrate compartment of the filter is complex (e.g., it has been demonstrated that predilution infusion in high efficiency systems results in a drop in dialyzer clearance compared to dialysis alone). We must assume that convection and diffusion do not simply add their effect but that a continuous interference between them is present [21]. We have not evaluated the role of a mixed therapy and purposefully have isolated the effect of diffusion to definitely characterize this, so our conclusions only apply for purely diffusive treatments (continuous hemofiltration) but not mixed modalities (hemodiafiltration) when some interaction should be expected. In these cases, when a high dialysate flow is prescribed, we think it is advisable to measure effluent saturation to ensure an adequate dialysis delivery. 

We have detected a significant difference in the urea and creatinine saturation; while the first one behaved as a good marker for saturation (all the data obtained from urea were consistent and narrowly distributed close to unity), creatinine showed a wide dispersion of data and is our conclusion that creatinine is not an adequate marker for dialysate saturation. We have no explanation for this finding but in part could be reflecting a problem with the determination of creatinine; our laboratory uses the Jaffe technique, known to show variations ranging from 5.3 to 27.4 *μ*mol/L [22].

We must acknowledge some biases in our results. The first one lays in the fact that we did perform the measures in a “laboratory” setting by standardizing the modality and not analyzing the patients who were really being treated. Consequently our conclusions do not apply when a mixed (convective-diffusive) therapy is performed. Our main objective was to detect the capability of the membrane to deliver a high dose of dialysis so opted for the use of this modality alone, limiting possible confounding factors and assuming the lose of external validity of our results.

An other limiting factor is the material employed; our results are only valid when CHD is performed with a 1.4 m^2^ AN69 membrane but, being this synthetic membrane the less efficient for diffusion, we believe that 50 mL/min can be considered a reasonable minimum for any other synthetic membrane employed in CRRT. 

Even though the number of measures was adequate to perform the analysis, the number of patients recruited is small and did not let us introduce patient related factors that hypothetically could have altered our results; so our conclusions must be understood as technically and not clinically oriented until more studies are published, but the similitude between our findings and those of Ricci et al. [17] is encouraging and support our conclusions.

We conclude that the AN69 membrane shows a higher diffusive performance than previously reported, up to 50 mL/min, covering efficiently the range of standard dosage for continuous therapies. 

## Figures and Tables

**Table 1:** Effect of anticoagulant regime on diffusive capability.

	Heparin	Epoprostenol	None	*P*
	18 (40.9%)	9 (20.5%)	17 (38.6%)	
Urea saturation	1.08 ± 0.08	1.07 ± 0.12	1.09 ± 0.09	ns
Creatinine saturation	0.89 ± 0.24	0.76 ± 0.22	0.87 ± 0.19	ns
*β*_2_-microglobulin saturation	0.33 ± 0.12	0.34 ± 0.11	0.34 ± 0.11	ns
